# Successful Composite Grafting of Glans Penis in Pediatric Traumatic Penile Amputation

**DOI:** 10.7759/cureus.22854

**Published:** 2022-03-04

**Authors:** Chun Kai Yew, Siti Fatimah Noor Mat Johar, Wan Azman Wan Sulaiman

**Affiliations:** 1 Reconstructive Sciences Unit, Hospital Universiti Sains Malaysia, Kota Bharu, MYS; 2 Plastic and Reconstructive Surgery, Universiti Sains Malaysia School of Medical Sciences, Kota Bharu, MYS

**Keywords:** pediatric penile trauma, composite graft, avulsion injury, glans penis, penile amputation

## Abstract

Traumatic penile amputation is a rare urologic emergency, more so in the pediatric population. It can result in significant consequences concerning function, appearance, psychological effects, and the overall quality of life. Immediate management should be initiated to salvage the amputated penis. We report a case of a four-year-old boy with a traumatic glans penis amputation caused by a sliding door accident. Successful composite grafting of the glans penis was done with an excellent functional and aesthetic outcome. This case highlights composite grafting as a viable option if microvascular replantation of penile amputation is deemed not feasible. Composite grafting is favorable if the grafted tissue is less than 2cm in size and the tissue is not crushed or contused.

## Introduction

Traumatic penile injury is uncommon as the penis is mobile and protected mainly by its position. Despite being a rare urologic emergency, it carries a significant functional and psychological impact on a patient’s quality of life [1]. However, there are only limited reports of traumatic penile amputation. The incidence of penile injury is likely under-reported, as patients do not seek treatment for psychological reasons or social embarrassment. In the pediatric age group, the etiologies of traumatic amputation of the penis differ from those in the adult population. The few reported causes include iatrogenic circumcision injuries, animal attacks, domestic abuse, motor vehicle accidents, zipper injury, and penile strangulation by hair [2].

We present a case of a four-year-old boy who accidentally amputated his glans penis with a sliding door and the usage of composite grafting in handling a pediatric glans avulsion injury.

## Case presentation

A four-year-old boy accidentally amputated his glans penis while playing with the sliding door. He was rushed to the local clinic, and the avulsed glans was wrapped with saline gauze and put into a plastic bag filled with ice. The time frame between amputation and reconstruction was seven hours.

Intraoperatively, the penile shaft was bruised and swollen with a hematoma in the prepuce. The remaining length of the urethra was filled with blood clots which were evacuated. The dorsal penile arteries, deep penile arteries, and dorsal veins were identified and patent.

**Figure 1:**
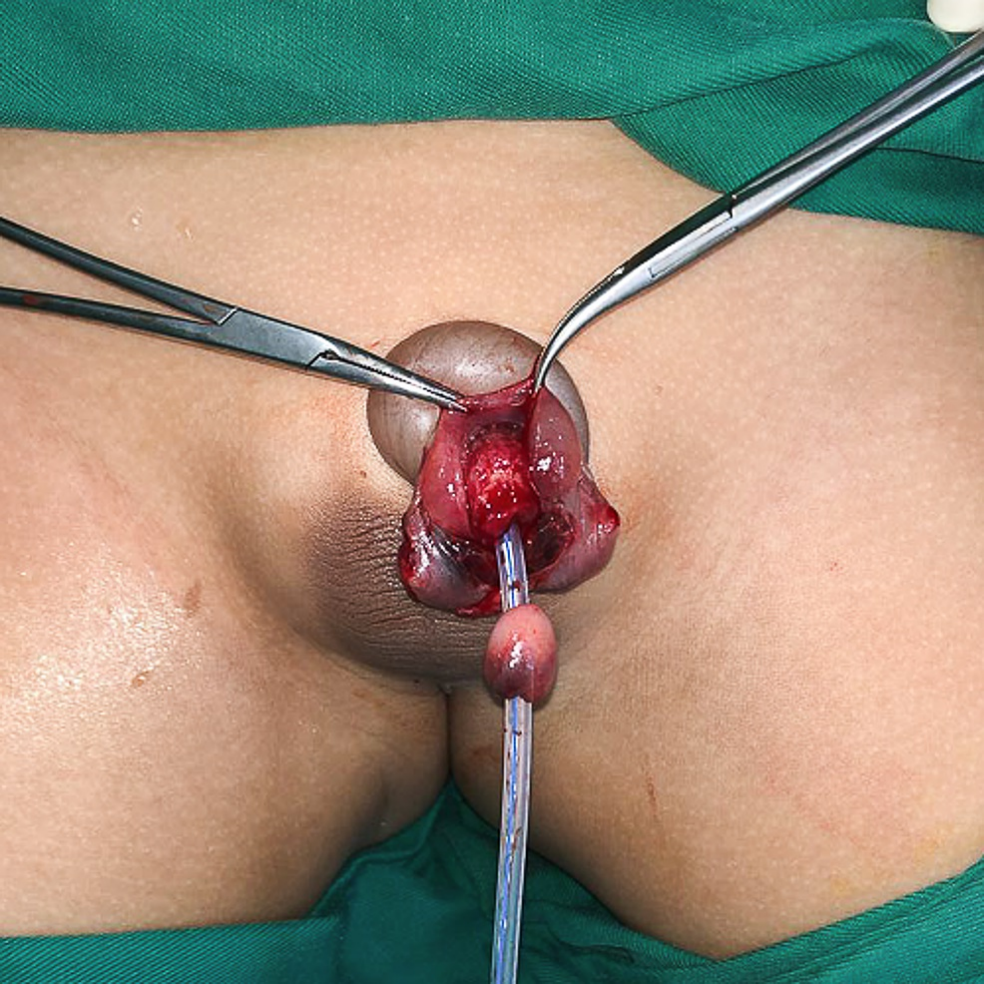
The amputated glans revealed the distal urethral meatus had avulsed along with the glans.

The amputated glans was measured at 1.6cm x 1.4cm in an oblique oval base with a height of 1.1cm. Examination revealed the distal urethral meatus had avulsed along with the glans, with bruising at the tip (Figure 1). The vessels on the glans were crushed with no suitable vessels for re-anastomosis.

**Figure 2:**
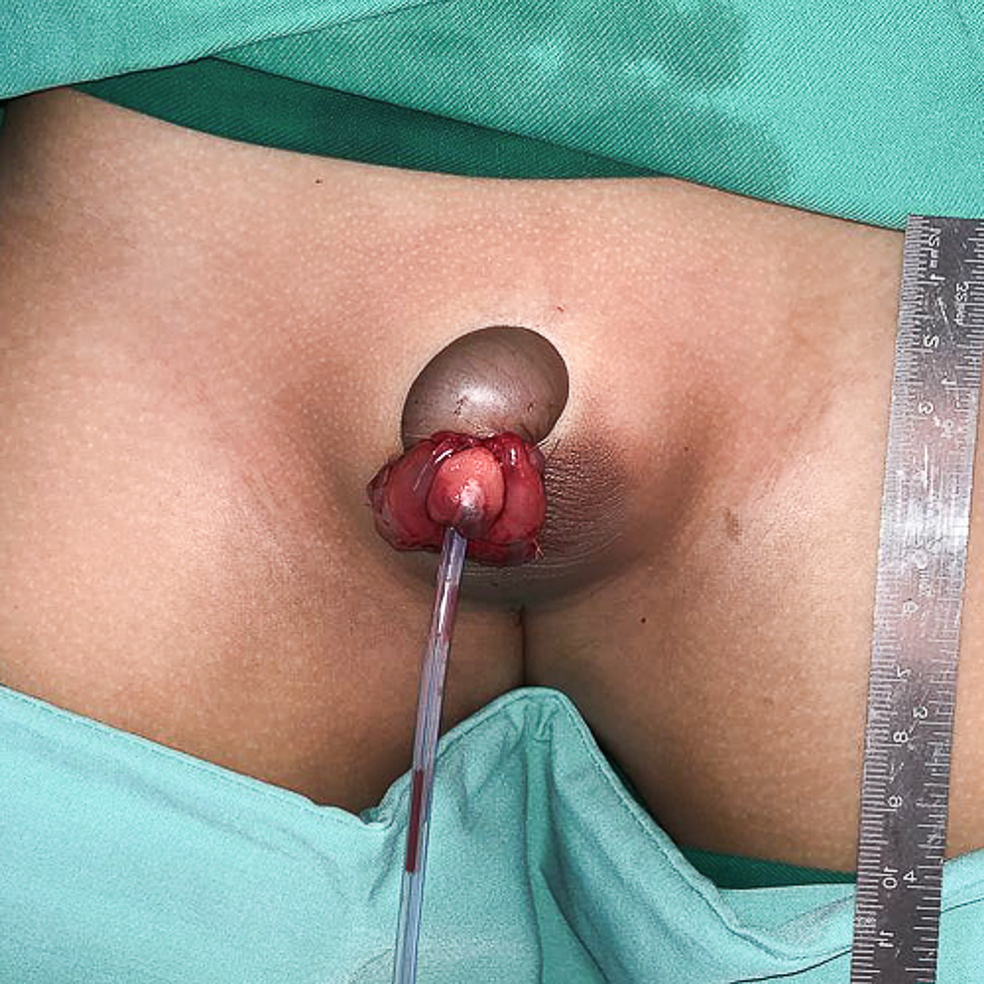
Composite grafting of the glans penis was done.

Composite grafting of the glans penis was done. A silicone urinary catheter was inserted, and the urethra was repaired with polyglactin sutures 7/0. The graft was anchored with polyglactin sutures 6/0 (Figure 2).

**Figure 3:**
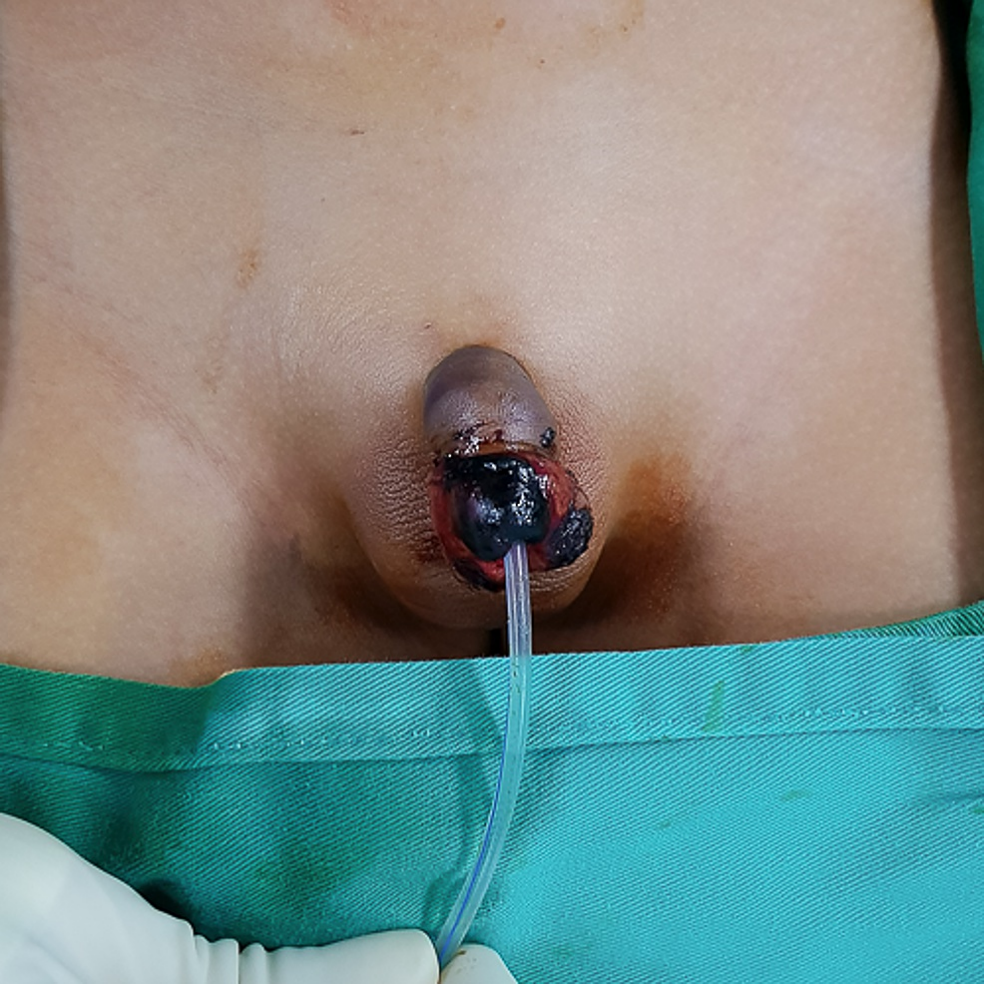
The glans became dusky over the tip with partial necrosis.

Post-operatively, the child was given parenteral antibiotics (cefuroxime and metronidazole) for two weeks. The glans became dusky over the tip with partial necrosis. It spread proximally progressively over the entire glans (Figure 3). By post-operation day 5, debridement of the necrotic areas of the glans was done, and a full-thickness skin graft harvested from the preputial skin was grafted over the raw wound.

The skin graft became necrotic as well and was debrided by post-operation day 10. The raw area was then covered with collagen dressing.

**Figure 4:**
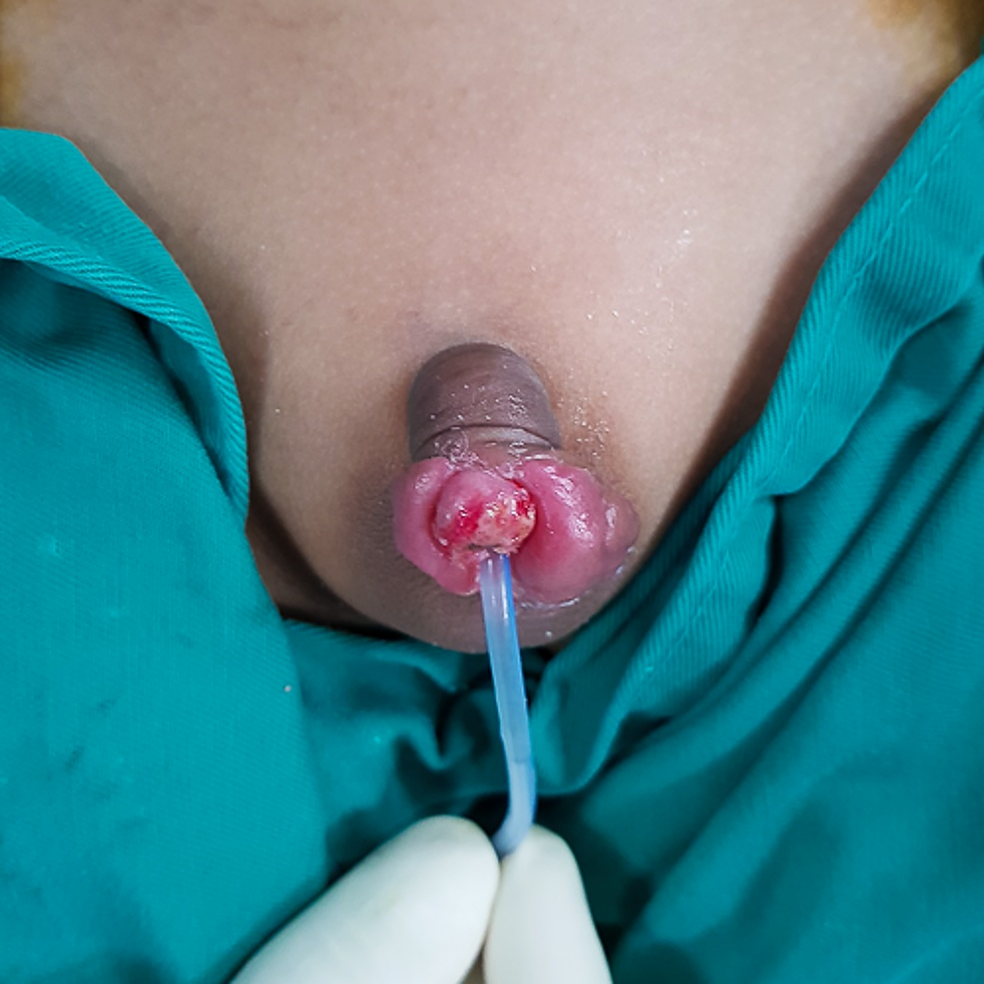
A healthy, viable composite graft of the glans penis was seen on post-operation day 21.

The collagen dressing was removed by post-operation day 21, which revealed a healthy viable composite graft (Figure 4). A retrograde urethrogram was done, which showed complete urethral integrity with no contrast extravasation and no evidence of urethral stricture. The child was discharged after the removal of the urinary catheter.

**Figure 5:**
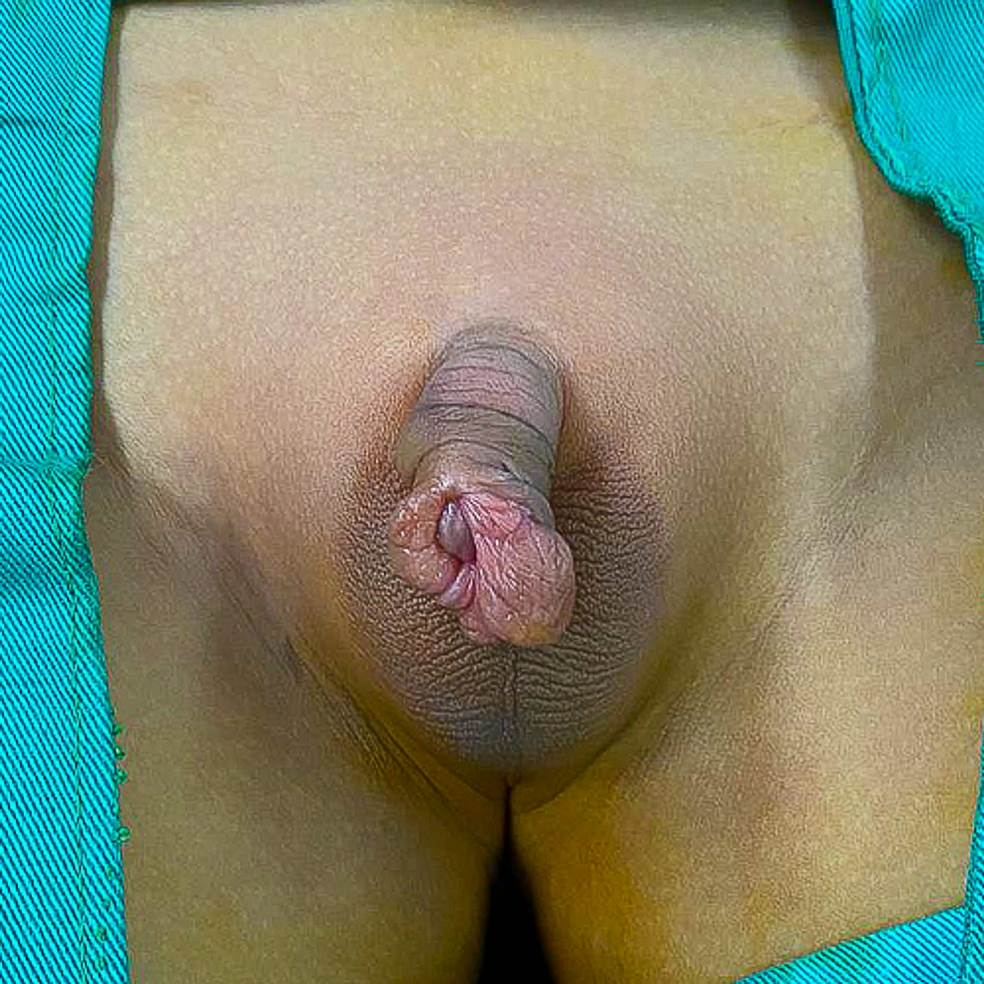
The glans penis composite graft was taken and viable six months post grafting.

The child came back for clinic review six months post grafting. The glans penis composite graft was taken and viable (Figure 5). He was able to pass urine well with a good stream and no complaints. Sensation over the glans was present but diminished.

## Discussion

Amputation of the penis is rare in the pediatric population. One of the most extensive series of penile trauma reported 64 cases, with the most common cause being circumcision injury (67%) and hair strangulation injury (16%), with 14% involving glandular trauma [2]. Assault has also been listed as a factor in this age group, either domestic animal attacks or sexual abuse [3,4].

The pediatric age group presents unique challenges, with patients on the younger spectrum unable to vocalize and seek prompt treatment. The reported case above was fortunate that his parent witnessed the event and brought him for treatment immediately. Even then, the logistic arrangements and transfer of care from the district clinic into a tertiary center resulted in an ischemic time of seven hours. Primary re-anastomosis surgery should be attempted if the injury occurred less than 24 hours [1], with previous studies highlighting that penile replant was successful when the total ischemic time was kept below 15 hours with a mean of seven hours [5]. There were also reports suggesting prolonged ischemic time up to 18 hours did not affect the outcome [3]. The patient above could have sustained more extensive tissue loss and necrosis if the surgery was delayed further.

Pediatric penile amputations also present with the increased complexity of the surgery and are technically more demanding due to the smaller organ and vessels. It was suggested in a case series that successful micro anastomosis of the penis at a prepubertal age is extremely difficult, if not impossible [6]. The nature, location, and severity of injury may compound the problem, and it ultimately depends on the surgeon’s skills and the facilities available. The first case of successful replantation via non-microsurgical techniques was reported in 1929, and subsequently, the first microvascular replantation was reported in 1977 [3,4]. Successively, microvascular replantation became the best practice for penile amputation in terms of survival and functions. However, the more distal penile injuries tend to be more technically challenging, particularly with vascular anastomosis due to the smaller vessels. In our case, the mechanism of injury of glans avulsion via sliding door led to a distally located amputation compounded with crushing of the vasculature, making microvascular replantation not feasible.

A graft is defined as a tissue transferred from the donor site without its blood supply. A composite graft is composed of two or more tissue components and therefore has a greater metabolic demand. Initially described by Konig in 1902 as composite auricular grafts for nose reconstruction, composite grafting has been popularized by Brown and Cannon, and the technique matured through time [7]. The increased metabolic demand dictates the size of composite grafts to be no more than 2cm, with no part of the graft being more than 1cm from the wound bed. This size limiting factor is favorable in distal penile amputations and pediatric patients. The avulsed glans for our patient measured 1.6cm x 1.4cm in an oblique oval base with a height of 1.1cm, making the glans suitable for composite grafting. As seen in our case, partial necrosis of the glans was expected due to the nature of the injury.

Additional methods for improving outcomes for composite grafts include inducing hypothermia over the graft with cooling and hyperbaric oxygen therapy [8]. Cooling can be utilized to slow down the biological demands of the graft until the graft becomes acclimated to the new environment [9]. However, this was not feasible to execute as the child was apprehensive. Hyperbaric oxygen therapy was found to be beneficial by elevating the partial pressure of oxygen in tissues. It increases fibroblast replication, capillary growth, collagen formation, and increased bactericidal function, ultimately improving graft survival [8,10]. Unfortunately, the hyperbaric oxygen unit was unavailable for the patient above.

The two critical areas of the function of the penis are voiding and sexual function. A successful penile replantation includes the ability to void without urethral stricture or fistula, absence of development of abnormal curvature, intact sensation, and recovering erectile function. In our patient, there are no concerns in terms of voiding. Further assessment is needed to follow up on his sexual functions in the future as he has diminished sensations over the glans. Circumcision will be suggested to remove the unequal preputial skin in the future.

## Conclusions

Penile amputation can result in significant consequences concerning function, appearance, psychological effects, and overall quality of life. Immediate and prompt management should be initiated to salvage the amputated penis. An urgent transfer to a center with microsurgical capabilities is essential, but composite grafting remains a viable option for specific cases if microvascular replantation is deemed not feasible. Composite grafting is favorable if the grafted tissue is less than 2cm in size and the tissue is not crushed or contused.
